# A method for estimating the deforestation timeline in rural settlements in a scenario of malaria transmission in frontier expansion in the Amazon Region

**DOI:** 10.1590/0074-02760170522

**Published:** 2018-07-23

**Authors:** Roberto Cardoso Ilacqua, Leonardo Suveges Moreira Chaves, Eduardo Sterlino Bergo, Jan E Conn, Maria Anice Mubeb Sallum, Gabriel Zorello Laporta

**Affiliations:** 1 Faculdade de Medicina do ABC, Setor de Pós-Graduação, Pesquisa e Inovação, Santo André, SP, Brasil; 2 Universidade de São Paulo, Faculdade de Saúde Pública, Departamento de Epidemiologia, São Paulo, SP, Brasil; 3 Secretaria de Estado da Saúde de São Paulo, Superintendência de Controle de Endemias, Araraquara, SP, Brasil; 4 The Wadsworth Center, New York State Department of Health, Slingerlands, NY, USA; 5 University at Albany, State University of New York, Department of Biomedical Sciences, School of Public Health, Albany, NY, USA; 6 Universidade Federal do ABC, Centro de Engenharia, Modelagem e Ciências Sociais Aplicadas, Santo André, SP, Brasil

**Keywords:** geographic information system, remote sensing technologies, malaria

## Abstract

The Malaria Frontier Hypothesis (MFH) is the current model for predicting malaria emergence in the Brazilian Amazon. It has two important dimensions, ‘settlement time’ and ‘malaria incidence’, and its prediction are: malaria incidence peaks five years after the initiation of human settlement and declines towards zero after an estimated 10 years. Although MFH is currently accepted, it has been challenged recently. Herein, we described a novel method for estimating settlement timeline by using remote sensing technology integrated in an open-software geographic information system. Surprisingly, we found that of the majority of the rural settlements with high malaria incidence are more than 10 years old.

Malaria was in the elimination phase in some endemic areas of the Amazon River Basin until 2017, when it re-emerged as a significant threat.^(^ ^1^ ^)^ This disease is a continuous threat to public health, especially in municipalities where the control program has been either reduced or discontinued for any length of time. This re-emergence scenario is more challenging because the potential for malaria transmission usually remains high due to environmental, social and economic determinants in the Amazon that favour the occurrence of the mosquito vectors and *Plasmodium* transmission. Approximately 128 thousand new malaria cases were reported in the Amazonian Region in 2016. The malaria incidence increased by 51% in 2017, up to 190 thousand new malaria cases. Of these, at least 127 thousand occurred in rural settlements or regions.^(^ ^1^ ^)^

The model for assessing the emergence of malaria in rural settlements is known as the Malaria Frontier Hypothesis (MFH).^(^ ^2^ ^)^ This model represents the temporal relationship between the colonization of a given area of Amazon forest and the emergence of the dynamics of malaria transmission.^(^ ^3^ ^)^ The MFH predicts that in rural settlements malaria incidence will peak at the beginning of the colonization process; then stabilize before reaching a low incidence rate usually ten years after the onset of colonization.^(^ ^4^ ^)^ The underlying mechanisms proposed for this pattern are chiefly related to improvements in both family income and community infrastructure over time that, in older settlements, could diminish or eliminate human-vector contact and improve access to malaria commodities, including health facilities, diagnostic tests and antimalarial drugs.^(^ ^4^ ^)^ Moreover, further studies have shown that host-parasite interactions can be modified depending on the time of colonization.^(^ ^5^ ^,^ ^6^ ^)^ The latter authors found that pioneer settlers have no immunity against malarial parasites, whereas older settlers can have partial immunity because of previous plasmodial infections. In other words, time of colonization can be either a risk or protection factor for malaria in rural settlements in the frontier expansion of the Amazon.

In contrast to the MFH, studies by Barros et al.^(^ ^7^ ^)^ and Barros and Honório^(^ ^8^ ^)^ found that old settlements are equally or more likely to have high malaria incidence (e.g., high parasite index) compared with a region that was recently inhabited. In this scenario, MFH can be a poor predictor of the dynamics of malaria transmission in the Amazon. Considering that the major goal of the United Nations Sustainable Development Agenda^(^ ^9^ ^)^ is the elimination of *Plasmodium falciparum* malaria in 2030, it is imperative to know whether current available models can accurately predict the emergence of malaria.

In this study we propose a method for estimating deforestation that addresses the potential association between the degree of deforestation and malaria incidence in rural settlements in the Amazon. The goal was to estimate time of colonization (in years) and percentage of forest cover in selected localities with landscapes of approximately 5-km^2^. Five-km^2^ is the approximate size of settler property within a forested matrix with potential larval habitats for malarial vectors and, ultimately, represents the spatial scale of the phenomenon of interest, i.e., the scenario of malaria transmission (e.g., landscape ecology textbook by Turner et al.^(^ ^10^ ^)^). Furthermore, deforested patches of this size have been shown recently to be significant drivers of malaria incidence across the Amazon^(^ ^11^ ^)^. We expected that all rural Amazonian settlements having high malaria incidence between 2015 and 2016 would be recently invaded (i.e., colonized) landscapes. However, we found the opposite: high incidences of malaria usually occurred in landscapes in which colonization and thus changes in natural landscapes had begun in the 1970s.

The localities were selected because of high annual parasite index (API ≥ 50) in 2015 or 2016 (Fig. 1). We selected 5-km^2^ landscapes, sorting them by forest cover category as follows: open areas (0-30%), moderately degraded (30-50%) and preserved (50-100%), having one replicate per category, totalling six landscapes per locality. A spatiotemporal regression model for the analysis of each landscape was conducted to estimate time of colonization. We assumed that 10% depletion of forest cover of a given landscape was indicative of the beginning of a human colonization process. Thus, we applied the 90% forest cover threshold for determining the start of colonization (t_0_ - starting time).

**Fig. 1:**
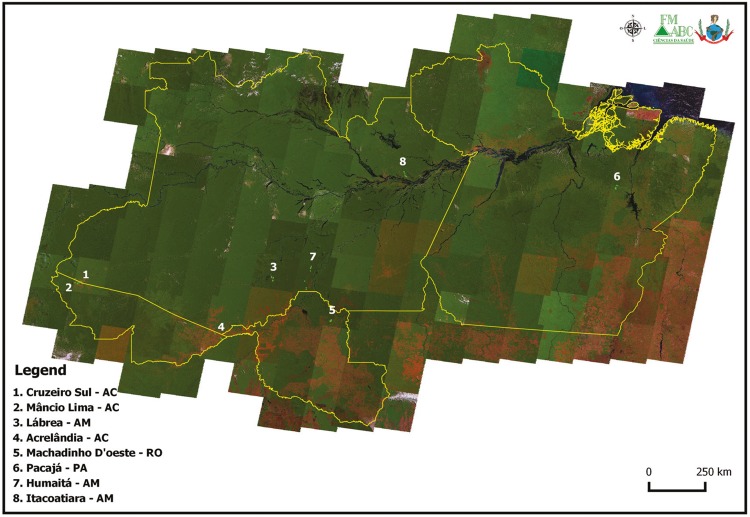
: study area. Sampled localities and landscapes in 2015-16, Amazon. The background is a RGB mosaic made from remote sensing images in 2011. Source: USGS, Landsat Project. Software: QGIS v. 2.16.2.

For the spatiotemporal regression model, we employed the geographic information system QGis v. 2.16.2 Nodebo (www.qgis.org) and the Landsat satellite imagery database (www.landsatlook.usgs.gov). Through the LandsatLook digital interface we accessed all the available databases of the Landsat satellites (Landsat 1-8). We acquired and used in this study imagery from Multispectral Scanner (MSS) 1972-1981, Thematic Mapper (TM) 1982-2011 and Operational Land Imager (OLI) 2013-2017 sensors. We used a combination of three bands (infrared, red, green), which showed a false colour effect in the landscape (Fig. 2). Next, the image was classified with the help of the Semi-Automatic Classification (SCP) plugin in the QGis (www.qgis.org). With this plugin we were able to perform a supervised classification with the satellite images as follows: preserved forest, dark green; exposed soil, yellow; urban soil, pink; rivers and lakes, blue; and unclassified sites, black (Fig. 2).

**Fig. 2:**
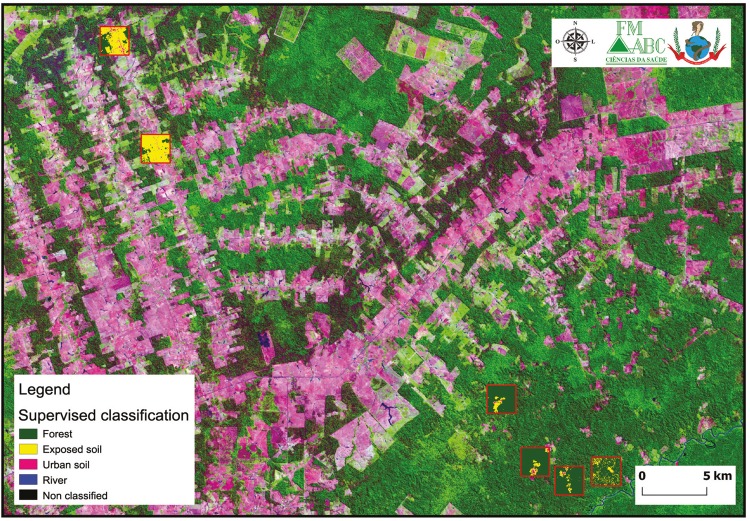
: image classification method. Supervised classification using a composite image of the locality of Acrelândia, in 2006. Source: USGS, Landsat Project. Software: QGIS v. 2.16.2.

For estimating the forest cover (%) in each landscape, we quantified the number of pixels classified as ‘forest’ divided by the total, times 100. This quantification was performed through two other plugins (Zonal Statistics and Group Stats), also available in the QGis. More information about the protocol of remote sensing methods for land use-land cover classification herein applied can be found in the Supplementary data - Protocol.

We repeated the same method of supervised classification per landscape in each locality in different years, going back from 2015 or 2016 (t_n_ - current time) to the year that each landscape forest cover reached the limiting threshold (> 90%) that represented the beginning of colonization. We then divided the landscapes into two categories: new settlement (≤ 10 years), and old settlement (> 10 years). This categorization was based on the prediction of the MFH, which states that new settlements are more likely to have high malaria incidence than old ones. Because all these landscapes were chosen in localities with a high annual parasite index for malaria (API ≥ 50), we expected to find more landscapes in the ‘new settlement’ category.

The results of the temporal regression per locality are depicted as follows: Cruzeiro do Sul-AC (Fig. 3), Mâncio Lima-AC (Fig. 4), Lábrea-AM (Fig. 5), Acrelândia-AC (Fig. 6), Machadinho d’Oeste-AM (Fig. 7), Pacajá-PA (Fig. 8), Humaitá-AM (Fig. 9), and Itacoatiara-AM (Fig. 10).

**Fig. 3:**
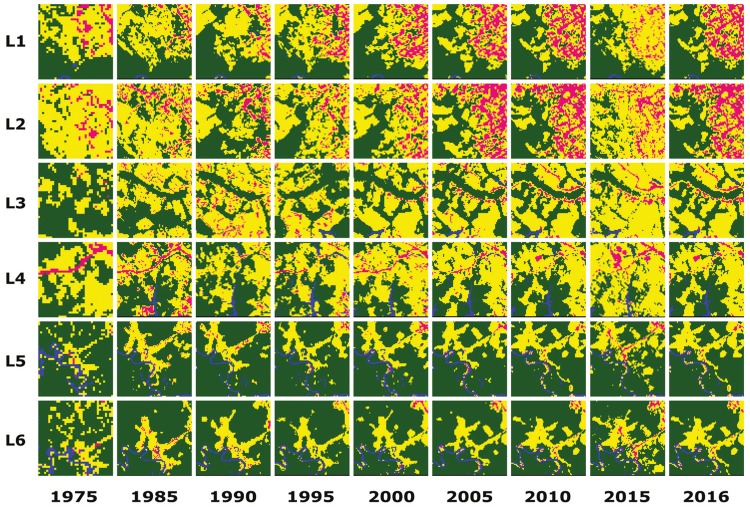
: Cruzeiro do Sul-AC. Temporal evolution of loss of forest cover. Landscapes (L1-L6) are each 5-km2. Parasite index for malaria in April/15: L1, 70.6; L2, 52.5; L3, 450.8; L4, 111.1; L5, 138; L6, 138. Source: Ministry of Health. Software: Inkscape v. 0.48.2.

**Fig. 4:**
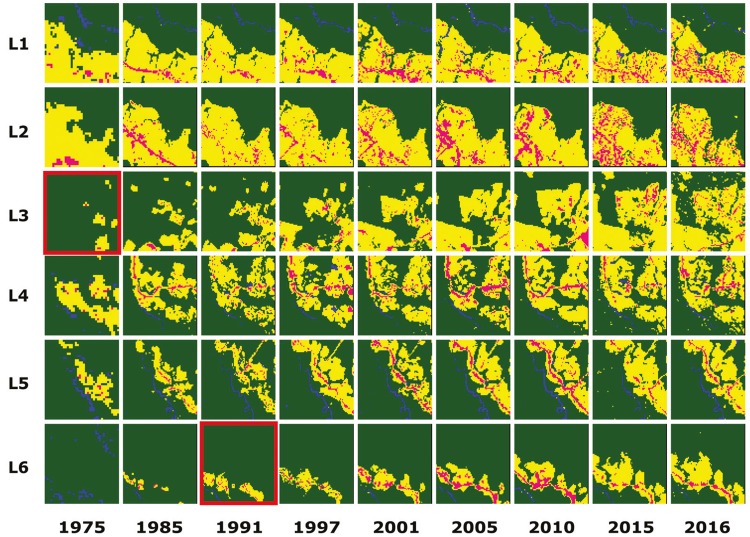
: Mâncio Lima-AC. Temporal evolution of loss of forest cover. Each landscape (L1-L6) is 5-km2. Parasite index for malaria in May/15: L1, 152.5; L2, 152.5; L3, 356.2; L4, 32.1; L5, 32.1; L6, 32.1. Source: Ministry of Health. Software: Inkscape v. 0.48.2. Red borders for pairs of location and time represent when and where the threshold of forest coverage < 90% was reached.

**Fig. 5:**
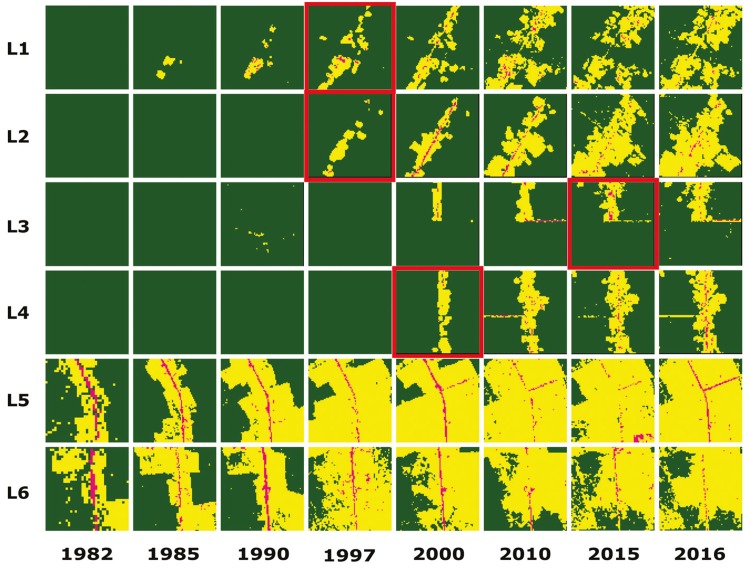
: Lábrea-AM. Temporal evolution of loss of forest cover. Each landscape (L1-L6) is 5-km2. Parasite index for malaria in July/15: L1, 447.9; L2, 447.9; L3, 173.5; L4, 173.5; L5, 447.9; L6, 447.9. Source: Ministry of Health. Software: Inkscape v. 0.48.2. Red borders for pairs of location and time represent when and where the threshold of forest coverage < 90% was reached.

**Fig. 6:**
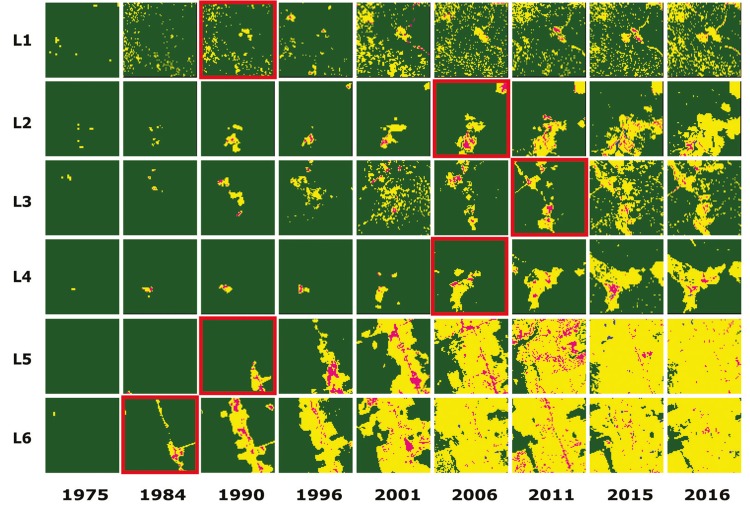
: Acrelândia-AC. Temporal evolution of loss of forest cover. Each landscape (L1-L6) is 5-km2. Parasite index for malaria in August/15: L1, 116.7; L2, 116.7; L3, 116.7; L4, 116.7; L5, 26.1; L6, 26.1. Source: Ministry of Health. Software: Inkscape v. 0.48.2. Red borders for pairs of location and time represent when and where the threshold of forest coverage < 90% was reached.

**Fig. 7:**
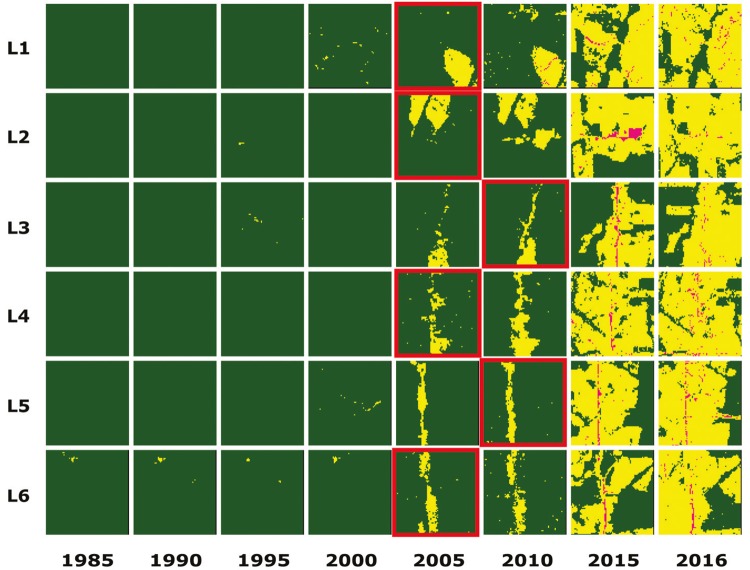
: Machadinho d’Oeste-RO. Temporal evolution of loss of forest cover. Each landscape (L1-L6) is 5-km2. Parasite index for malaria in October/15: L1, 185.2; L2, 150; L3, 150; L4, 150; L5, 230.8; L6, 185.2. Source: Ministry of Health. Software: Inkscape v. 0.48.2. Red borders for pairs of location and time represent when and where the threshold of forest coverage < 90% was reached.

**Fig. 8:**
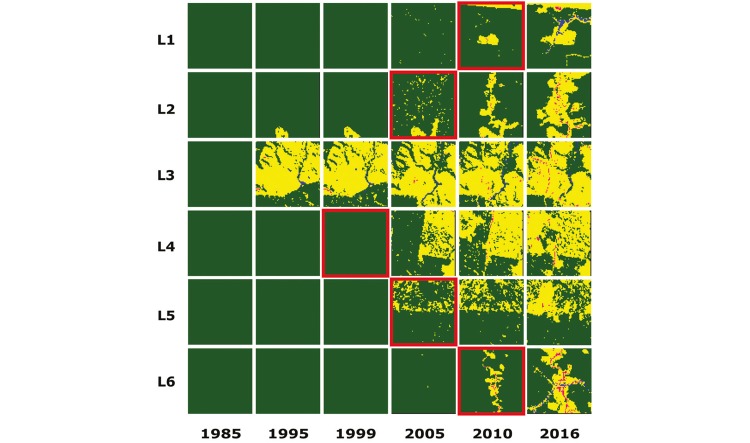
: Pacajá-PA. Temporal evolution of loss of forest cover. Each landscape (L1-L6) is 5-km2. Parasite index for malaria in April/16: L1, 53.6; L2, 53.6; L3, 53.6; L4, 16.3; L5, 26.5; L6, 53.6. Source: Ministry of Health. Software: Inkscape v. 0.48.2. Red borders for pairs of location and time represent when and where the threshold of forest coverage < 90% was reached.

**Fig. 9:**
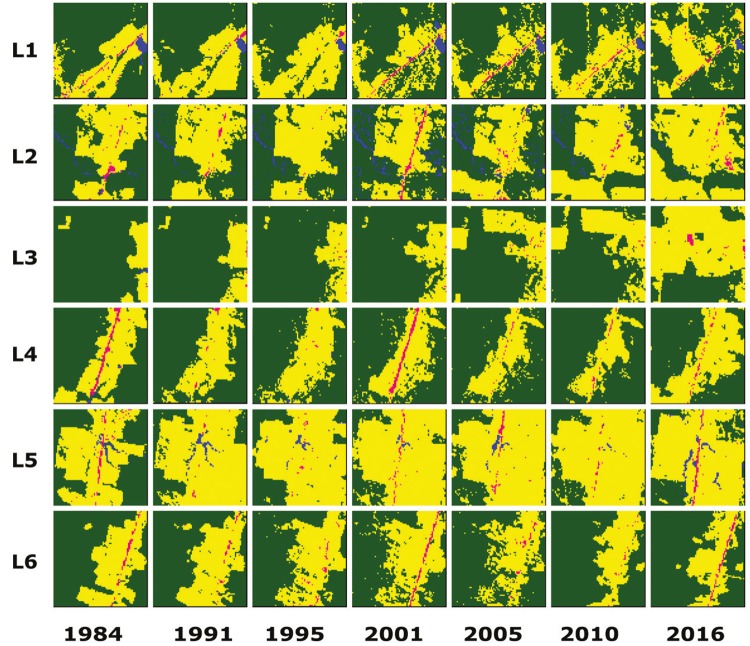
: Humaitá-AM. Temporal evolution of loss of forest cover. Each landscape (L1-L6) is 5-km2. Parasite index for malaria in July/16: L1, 191.8; L2, 31.6; L3, 277.8; L4, 31.6; L5, 31.6; L6, 31.6. Source: Ministry of Health. Software: Inkscape v. 0.48.2.

**Fig. 10:**
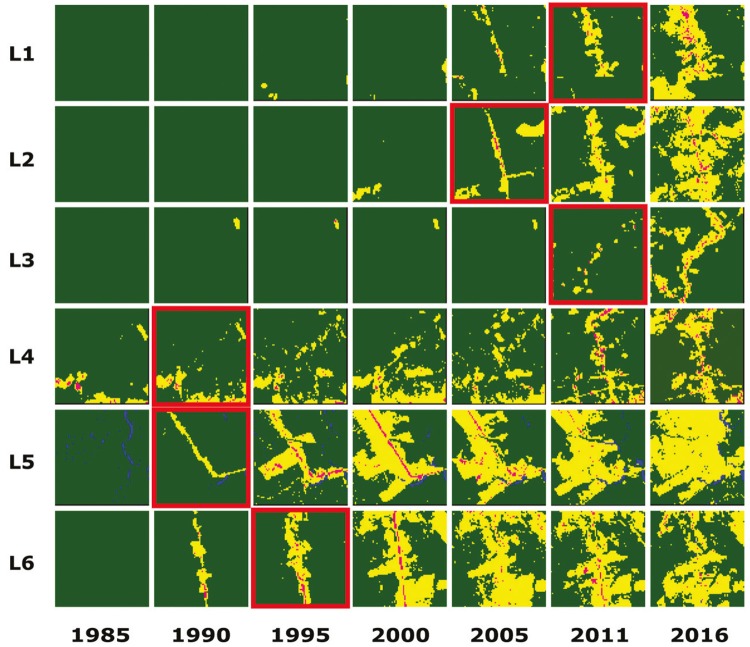
: Itacoatiara-AM. Temporal evolution of loss of forest cover. Each landscape (L1-L6) is 5-km2. Parasite index for malaria in November/16: L1-L6, > 50. Source: Ministry of Health. Software: Inkscape v. 0.48.2. Red borders for pairs of location and time represent when and where the threshold of forest coverage < 90% was reached.

Out of 48 landscapes studied, eight (16.67%) were categorized as ‘new settlements’ with time of colonization ≤ 10 years, while the remaining 40 (86.33%) corresponded to ‘old settlements’ (time of colonization > 10 years). A test of independence for a potential association between settlement age and parasite index was performed for the 48 malaria landscapes. The outcome of this test was statistically insignificant, and does not support the prediction that malaria incidence peaks more generally in recently invaded rural settlements of the Brazilian Amazon (Table). Therefore, we propose an alternative model based on the forest fringe model (Barros et al.^(^ ^7^ ^)^; Barros and Honório^(^ ^8^ ^)^). The main assumption of the alternative model is that landscape variables (e.g., fragmentation thresholds) govern the dynamics of malaria transmission in the Amazon. According to the forest fringe model, malaria emergence is most likely to happen when the landscape is fragmented, because this scenario provides larval habitats in forest patches near anthropogenic areas where vector-host contact occurs. Through logical deduction, malaria prevention would be possible based on landscape thresholds, as follows: either the forest environment is preserved (> 90% forest cover) or it is efficiently transformed into an urban/rural area (< 10% forest cover) with essential infrastructure. But, at one extreme, Brazil’s Forest Code is not respected^(^ ^12^ ^)^ and at the other, the anthropogenic matrix is not adequately improved^(^ ^13^ ^)^, leaving most human settlements comprised of fragmented landscapes (70-30% forest cover) where malaria emergence will be a perennial challenge for public health. Landscape thresholds for malaria emergence can be of practical value in malaria control and elimination scenarios.

**TABLE:** Contingency matrix 2 by 2 with Parasite Index for Malaria vs. Settlement Time in landscape (n = 48)

Settlement time	Parasite index for malaria	

	≥ 50 cases per 1,000	< 50 cases per 1,000
New (≤ 10 years)	8	0
Old (> 10 years)	29	11

Fisher’s exact test for count data: alternative hypothesis, new settlements are associated with higher malaria incidence (malaria frontier hypothesis). Decision: accept the null hypothesis (there is no association); p-value = 0.1704.

Nevertheless, this study has some limitations. One is related to the spatial resolution (~ 60-m) of multispectral scanner satellite imagery obtained in 1972-1981. The use of those images with a resolution that is lower than that of recent images might have caused inaccurate estimations of forest cover. Although we were not able to identify the scale of this inaccuracy, we believe that it could range between 5-20%. Qualitatively speaking, the conclusions would be similar, because we had access to higher-resolution thematic mapper images (30-m), from 1982 on. The second limitation concerns human mobility.^(^ ^14^ ^)^ Migratory waves augment both susceptible and infectious hosts, thus increasing malarial transmission.^(^ ^15^ ^)^ However, considerations of such data were beyond the scope of the present study.
