# Supplementary material for: A method for estimating the deforestation timeline in rural settlements in a scenario of malaria transmission in frontier expansion in the Amazon Region
Source: Mem Inst Oswaldo Cruz. 2018 Jul 23;113(9):e170522. doi: 10.1590/0074-02760170522 (PMC6055538; doi:10.1590/0074-02760170522)
Supplement: Supplementary file 1 [file 0074-0276-mioc-113-9-e170522-Suppl01.pdf]

Roberto Cardoso Ilacqua

QGis Handbook for Supervised Classification of Areas

Santo André

2017

Roberto Cardoso Ilacqua

QGis Handbook for Supervised Classification of Areas

This manual was designed to assist the students of the Environmental Health Management course of the Faculty of Medicine of ABC, to perform the remote sensing from the Landsat^{MT} satellite images, integrated in the QGis software, for the classification and quantification of land use and land cover

Santo André

2017

Summary

Introduction 4
Introduction to installation 5
Land Use-Land Cover (LULC) Protocol Steps 6
Protocol Flowchart 7
Understanding all steps of the Process 8
Step-by-Step Protocol 11
1. Determining the location under study 11
2. Collecting data as coordinates and area of study location 11
3. Transforming the coordinate data and area of the locality into a Shapefile file 11
4. Folder template (see Figure-1) 11
5. Downloading a Landsat satellite image at www.landsatlook.usgs.gov. 11
Figure-1. Folder template. 12
6. Reflectance of bands 12
7. Georeferencing of bands 14
8. Total RGB of the scene 15
9. Cutting out the areas under study 17
10. Cutting out RGB 18
11. Loading the SCP (Semi-Automatic Classification) plugin to start sorting 19
12. Getting Started 20
13. Starting the Training (making the signatures of the pixels) 21
Defining the colors of each class 22
Setting the colors of the Macro Class 22
Defining the algorithm for Classification 22
14. Starting and Saving land use and land cover rating 23
Saving the rating 23
15. Quantifying land use and land cover 24
16. Transforming the Raster from sorting into a Shapefile 25
17. Loading Plugin Complement Statistic by Zone 26
18. Loading the Group Stats plugin 27
19. Adding the signatures according to the color of each 28
20. Calculating the percentage of land use land cover 29
21. Using the printing composer 30

Introduction

The protocol described below is a complete manual for performing the download and treatment of sattelite scenes, such as cutting areas under study, supervised classification and pixel quantification of land use land cover categories.

Remote sensing today is a widely used process in research around the world for those who wish to classify and quantify land use and occupation over time in urban or forest areas.

This protocol uses the QGis software, which is freely accessible, and satellite data (images) are obtained through the Landsat satellites through the LandsatLook platform.

This protocol was initially developed to observe and quantify the temporal dynamics of forest cover in the Amazon forest and to relate it to the dynamics of malaria transmission in the Brazilian Amazon.

Introduction to installation

As a first step, you must install the software and plugins used in this protocol.

To download the free access software QGis go to http://www.qgis.org and preferably download the latest version.

After downloading QGis (latest version), you must install the plugins. On the top command bar, click on the Add option and select Manage and Install Add-ons, select and install one plug-in at a time.

First the SCP plugin Semi-Automatic Classificatication, then the Plugin Statistic by Zone , and finally the Group Stats plugin .

Land Use-Land Cover (LULC) Protocol Steps

1. Determining the location under study.

2. Collecting data such as coordinates and study area.

3. Transforming the coordinate data and the local area into a Shapefile file.

***** *Data above provided by the researcher responsible for this study* (GZL).

4. Folder template (see Figure-1).

5. Download the scenes from the Landsat satellites.

6. Reflectance of bands.

7. Georeferencing of bands.

8. Full RGB of the scene.

9. Cutting out the areas under study.

10. Cutting out RGB.

11. Loading the SCP (Semi-Automatic Classification) plugin.

12. Loading the RGB from the clipping into the SCP plugin.

13. Starting the training (make signatures of the pixels).

- Defining group and subgroup of signatures.
- Determining the colors of each group and subgroup.

14. Starting and saving the land use and land cover rating.

15. Quantifying land use and land cover.

16. Transforming the Raster from sorting into a Shapefile.

17. Uploading the Statistics plugin by Zone.

18. Loading the Group Stats plugin.

19. Adding the signatures according to the color of each one.

20. Calculating the percentage of land use and occupation.

21. Image Composer.

Protocol Flowchart

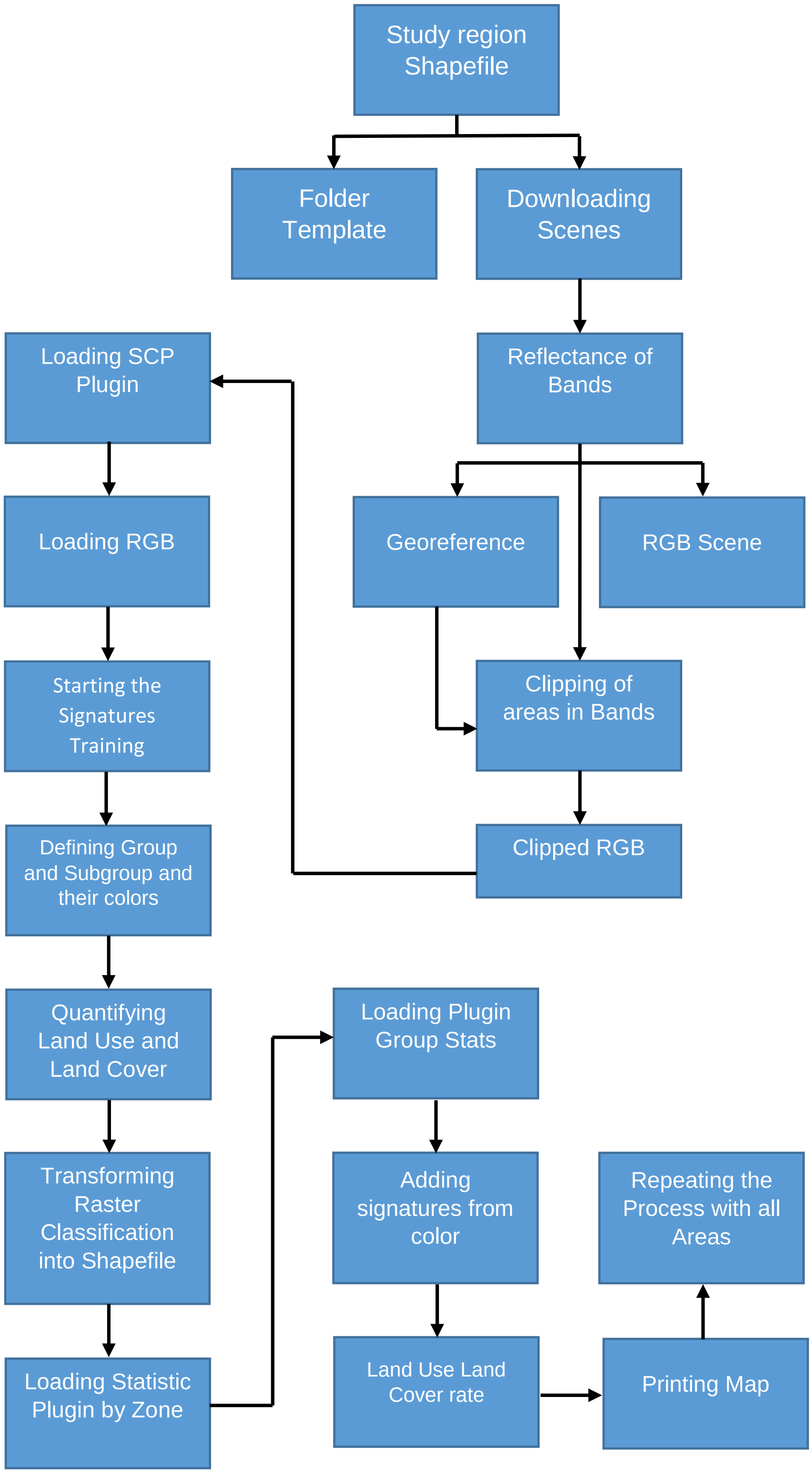

Understanding all steps of the Process

Determining the location under study

The study sites should be chosen according to what you want to study, a location with a high rate of zoonotic diseases for example.

Collecting data as coordinates and area of study location

Fieldwork made in the study areas, using equipment such as a GPS, to obtain the coordinates of each area under study, which can also be done by remote sensing.

Transforming the coordinate data and area of the locality into a Shapefile file

*Data above was made available by the researcher responsible (GZL) for the study, including the Shapefile of the study area.

Folder template (see Figure -1)

These folders are very important because they will be used to organize all the information obtained during the process of treatment and classification of each area.

Downloading the scenes from the Landsat satellites

Use the LandsatLook platform at www.landsatlook.usgs.gov, which is easy to manipulate, to download the scenes in which the study areas are contained.

Reflectance of bands

The band reflectance process is an algorithm that helps to improve the sharpness of the bands thus improving the final RGB product.

Georeferencing of bands

The process of georeferencing of the bands is a process that is only used when the scenes are out of position, which means that the study area is outside the correct location, even though it is in the same projection.

Total RGB of the scene

The total RGB of the scene is used to merge all the bands of the scene, allowing the manipulation of these bands, being able to compose scenes of different spectra of light that will provide colored images like the natural color formed by the Bands 1-2-3, or false color formed by Bands 5-4-3, which is used in this protocol.

With this protocol, images of all satellites available on the LandsatLook platform can be utilized, but each satellite has different equipment for capturing the images, this causes each one to have different bands, altering the sequences of the bands used to merge the RGB composite.

*Obs. Satellite images available on the LandsatLook platform and their bands sequences to form the RGBs are:

Landsat 1-3; Bands: 4-5-6-7; RGB: 4-5-6-7; RGB False Color: 6-5-4.

Landsat 4-5; Bands: 1-2-3-4-5-6-7; RGB: 1-2-3-4-5-7; RGB False Color: 5-4-3

Landsat-7**

Landsat-8; Bands: 1-2-3-4-5-6-7-8-9-10-11-BQA; RGB: 2-3-4-5-6-7; RGB False Color: 5-4-3.

** The Landsat-6 satellite exploded when it was launched; Landsat-7's main camera sensor was damaged when the satellite was already in orbit. The only product available by Landsat-7 is the panchromatic image, which was not used in this protocol.

Cutting out the areas under study

This technique is used to cut the area of study of the total scene, so the area of study becomes evident, facilitating its classification and quantification.

Cutting out RGB

With the bands that were treated in the previous protocol, we merge them into a RGB composition, allowing the manipulation of these bands by composing a scene with the natural color (Bands 1-2-3), or false color (Bands 5-4-3) and used in this protocol.

Loading the SCP (Semi-Automatic Classification) plugin

This tool and its algorithm are provided by the plugin itself, and serve to classify the land use land cover, among other things.

Loading the trimmer RGB into the SCP plugin

To start the classification of the land use land cover is necessary to load the RGB composite of the study area in the SCP plugin.

Starting the training

After loading the RGB composite in the SCP plugin, the training data is applied, for training the plugin to classify land use land cover.

Defining group and subgroup of signatures

Training is based on signatures, which are polygons of each type of land use land cover, sorted into groups, such as soil, water, and subgroups, such as exposed soil, urban soil, river and lakes.

Determining the colors of each group and subgroup

According to the signatures of the groups and the subgroups, the colors of each will be defined, in a suggestive way, for a better contrast in the visualization of the land use land cover.

Starting and saving the land use and land cover rating

After saving the training, the classification of the area starts. The algorithm of the SCP plugin interprets each signature and classifies according to land use land cover. It must be saved in the classification folder of each area individually.

Quantifying the land use land cover

With the classification finished and saved in the folder correctly, each element of land use land cover must be quantified in percentages. It is necessary two more plugins, also available in the QGis software: Complementary statistics by zone and Stats Group.

Transforming the Raster's classification into a Shapefile

The final product of the classification is a Raster, but Raster can not be quantified. So it must be transformed into a vector file format.

Statistical plugin by zone

This plugin allows quantifying the pixel number of each signature, using count, sum, average, maximum and minimum per a given area (zone).

The Group Stats plugin

This plugin will allow us to work on the zones determined in the statistical plugin by zone.

Adding the signatures according to the color of each

After running the two plugins, add the signatures according to the color.

Calculating the percentage of land use land cover

This calculation will provide the amount of pixels of land use land cover in percentage, to facilitate the interpretation of the data.

Using the printing composer

The print composer is for printing the classification as an image.

Step-by-Step Protocol

1. Determining the location under study

2. Collecting data as coordinates and area of study location

3. Transforming the coordinate data and area of the locality into a Shapefile file

*****Data above made available by the researcher responsible for the study (GZL), including the Shapefile of the study area.

4. Folder template (see Figure-1)

5. Downloading a Landsat satellite image at www.landsatlook.usgs.gov.

The platform of the site has easy access and is self-explanatory. It will be demonstrated here step by step to download the scenes, when entering the platform. The image is a topography filter, it should be changed to real image, click on the Basemap gallery button, choose the image option, first option on the left side. Now the image is in natural color, closer to reality.

Click on the Search button and enter the name of the locality, city, municipality or neighbourhood. As an example, it was used the municipality of Mâncio Lima, in the state of the Acre, in Brazil. Then, the platform immediately searches the locality.

Click on the Load Images button to select the desired satellite and the maximum cloud coverage. In the example, the Landsat 4-5 satellite, which covers images from 1982 to 2011, was chosen.

When choosing the satellite, click on Show Images and the platform will search in your database the images with the desired profile.

After searching the platform database, the range of available images will be displayed.

For a better understanding of the scope of each scene, it is necessary to use a layer superimposed on the map that delimits each scene. Then, click on the Map Overlay button and choose WRS2 Path/Row Overlay (blue lines).

Blue lines appear on the entire map. These lines form the perimeter of each scene. In the center there is a number, in this case 5/65, which represents the position of the scene on the terrestrial globe. In the Display Image choose the option Active Data Only and not Mosaic, so only the active images for the locality searched will appear on the screen.

After choosing the scene you want to download, click on in the Export Images button , and then click on Export. The platform will export all available images.

After the end of the export process, click on Hide Metadata, a window where all available images will appear, ant then choose the scene according to the desired date on the left side of the window. Following, the table after the date will have important information such as the number of the scene in relation to the terrestrial globe, and next to it has the percentage amount of clouds on the scene. Click on Full Product Download, and then the interface to download the scene will be opene. Click on the green arrow on the right side of the screen next to the red X.

Several products are available. For this protocol we used the product Level-1 Geo TIFF Data Product. When download is complete, save information in the Raw Data folder.

Figure-1. Folder template.

It is necessary to make a folder template so that you can organize and save the information produced during the application of the protocol.

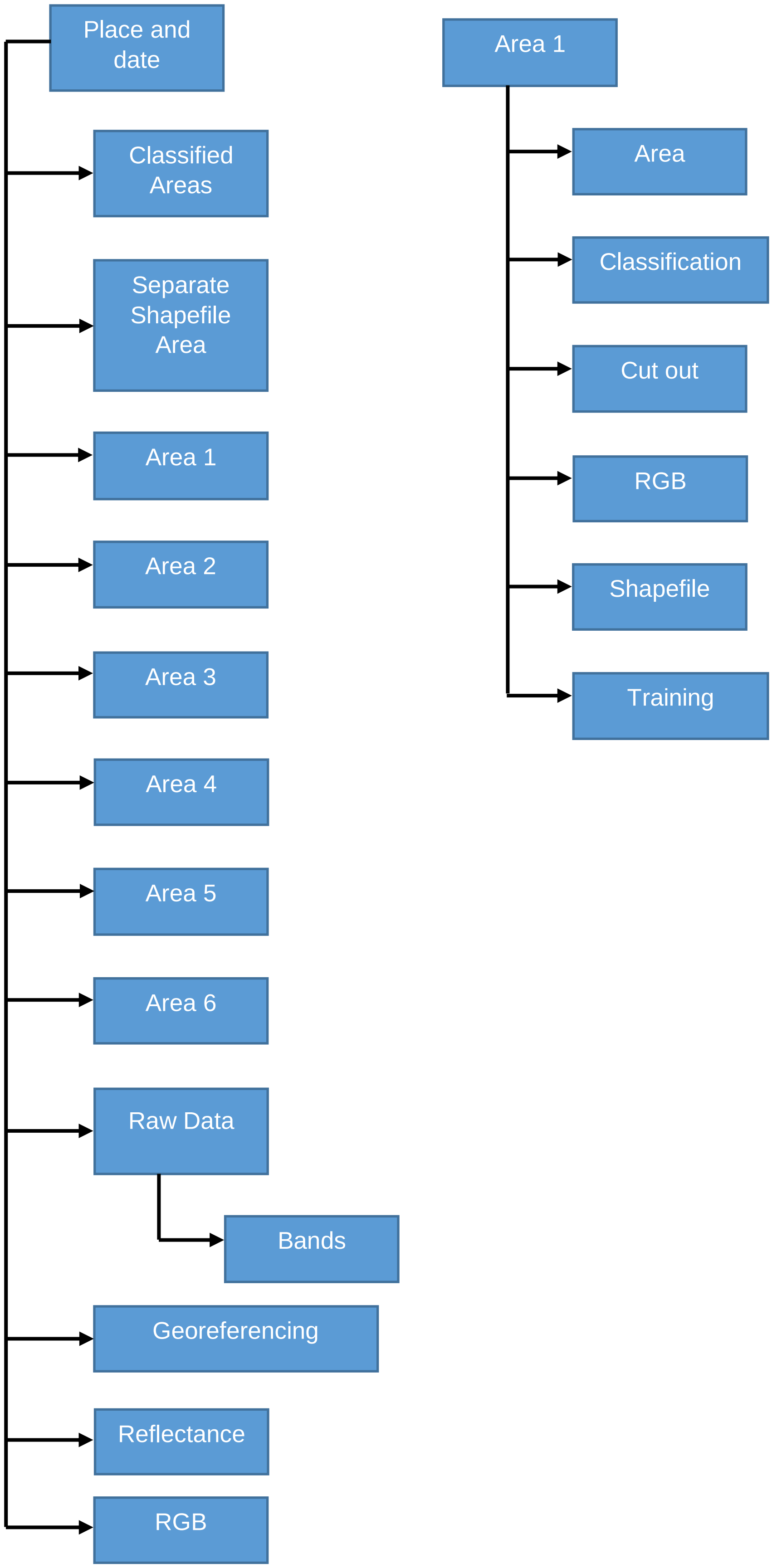

6. Reflectance of bands

This process helps improve the sharpness of the bands. Click on Semi-Automatic Classification (SCP), to start the process.

In the window that opened click on Preprocessing Preprocessing which is pre-procedure.

There are several tool tabs available. The Landsat tab will be used Landsat .

Click on the select a directory button to load the folder with the bands in the scene, open the Raw Data folder, and select the Bands folder.

After loading the Bands folder, you must load the MTL file by clicking on the Open a file key, the MTL file must be saved in the Raw Data folder.

After all the information loaded, just click on the Run key in the lower right corner.

Select the Reflectance folder, so the new bands are saved. This process lasts a few seconds.

At the end of the process the new bands are loaded automatically. Now the bands have an RT prefix.

7. Georeferencing of bands

Georeferencing is only used when the bands are slightly out of the correct position in relation to the Shapefile of the study area.

First load an RGB that is correct in relation to the study area by clicking on the Raster key.

Look for common dots between the correct RGB and the bands that are out of the correct position. Click on the Georeference button.

When you open the georeferencing panel, click on the Open Image button, and open the Reflectance folder to select one of the Bands, which are outside the correct location. With the band loaded in the georeferentiator, click on the Add Points button.

Choose common points of the band to be corrected and the correct RGB. When marking a point, the geo-referer will ask if you would like to enter the coordinates or mark a point from the map on the screen, choose mark from the map on the screen and check the same point that was marked in the Band, in RGB.

By marking the same point on the map screen the coordinates are saved automatically, the point is marked in the Band, and the coordinates are saved.

This process must be repeated at least three times or more, preferably, and the points form a triangle, so that the georeferencing becomes more accurate.

After marking the three points in the wrong band and the correct RGB, click on the Start button Georeferencing. The system will ask to set the Transformation type, click on OK.

When you open Transformation Setup, click on Output Raster and select the Georeferencing folder to save the new Band.

The new band will have the same band name and the only difference that the new band will have is a modified suffix, click on Save.

The transformation settings window will open again with the address where the new modified band will be saved, click on OK.

After the modification, make the modified RGB and compare with a correct RGB to see if they are overlapped correctly.

After marking the three points, click on the Save GCP points button, so that these saved points are used in the other bands.

To use the saved points in the other Bands, simply click on the Load GCP points button.

Open the folder where the points file is saved, click on the file and the points will be loaded immediately.

Soon after will appear the message GCP file loaded successfully.

8. Total RGB of the scene

Before starting to do the RGB composition, you have to know which satellite provided the scene, since each satellite has its own combination for RGB fusion. In this protocol, images of the Landsat 4-5 satellites are used; with the following merging scheme: RGB bands 1-2-3-4-5-7 are used.

Click on the Raster key to load the Bands, open the Reflectance folder, and select all Bands in the folder.

When opened, the Bands appear open on the left side of the screen.

Click on the Close All button, above the legend, the Bands are in descending order from Band-7 to Band-1, the order must be increasing and reversed from Band-1 to Band-7.

For the RGB merging process, it is necessary to remove the sixth Band because it is not part of the process.

To delete Band-6, select the band to be deleted and click on the Remove Layer/Group button, and the system will ask (Remove 1 entry from the legend?).

Click on the Semi-Automatic Classification button, the SCP plugin window will open, then click on the Band set tab Band set .

Click on the Refresh list key to the right of the window, this button will load the bands that are in the legend, in the same order.

Click on the Select all key, so that all bands are selected, then click on the Add Band to Band button on the right side of the window.

The selected bands go to the lower window in the same order, select in the lower left corner of the window, then Create a virtual Raster of band set option to create the RGB.

Above the Create a virtual Raster of band set option, there is a Quick wavelength settings window.

It will open a window with several satellite options; choose the one you are working at the moment.

With the option chosen, select Landsat 4-5 TM [bands 1, 2, 3, 4, 5, 7].

Now the information is loaded, just click on the Run key on the lower right side to save and start the RGB merge process.

Save to the RGBs folder, and the process will start automatically after a few seconds.

RGB is presented in natural color, composed by the combination of Bands 1-2-3.

This protocol uses the combination of false color, position the mouse cursor over the RGB and click on the right button, a quick access menu will appear, click on properties.

You will see the properties of the Raster you just merged. Open the Style tab on the left side of the window, see fields Red Band, Green Band, and Blue Band appear, which are filled by Bands 1-2-3, respectively, for false color the field of the Red Band is Band-5, Green Band = Band-4, and Blue Band = Band-3.

Open the Load Min/Max Values window; select the Cumulative Count Cut option, and then load.

The Min and Max information for each Band will change, and the image behind the window will change color.

To finish, click on Apply in the lower right corner of the window, and then Ok. The RGB is false color, with the combination of Bands 5-4-3 in that order.

Approaching the image a little, it is observed the elements of land use land cover in different colorations and shades.

9. Cutting out the areas under study

The cut of the study areas allow working only with the areas of interest. To start the process, you must first load the bands you want to cut the area under study. To do this click on the Raster key, open the Reflectance folder and select all available in the folder.

When loading the bands that were presented in a descending order from Band 7 to Band 1, Band should be placed in ascending order from Band 1 to Band 7. Now the Shapefies of the areas under study will be used to cut out the areas of interest. Click on Vector , then on Search, open the folder of Separate Areas and select files with the extension .shp.

After the successfully loaded shapefile, open the SCP plugin, clicking on the Semi-Automatic Classification button and in the plugin window click the Clip tab Clip multiple rasters

Click on the Refresh list button on the right side of the window to load the bands, then click on the Select all button, so that all the bands are selected.

Select the Use Shapefile for clipping option, to make the cut using the Shapefile, click on the Refresh list button on the bottom right of the window, to load the shapefile. It will open a list with all the Shapefile loaded, select the landscape 1.

Click on the Run button in the lower right corner to start the process, and choose the Cut folder in the Area-1 folder, and save it.

Click again on the Refresh list button on the bottom right of the window, and select the shapefile-2, click the Run button in the lower right corner to start the process.

Choose the Crop folder within the Area-2 folder, and save it.

Make this process as many times as necessary according to the number of areas under study.

10. Cutting out RGB

To start loading the clippings of the area you want to sort, click on the Raster key.

Click on the Clipping folder and select all Bands in the folder.

Put the Bands in ascending order and remove the Band-6.

To remove Band-6, select Band-6 and click the Remove Layer / Group button.

The system will ask if you want to Remove 1 legend entry, click on OK.

Open the SCP plugin to start merging RGB.

Select the Band set tab Band set .

Click on the Refresh list button on the upper right side of the window to load the Bands, and then click on the Select all button. Now click on Add band to Band set button on the right side of the window, and the Bands will be loaded to the window underneath.

Open the Quick wavelength settings window, and choose the satellite according to which you downloaded the scene, for that Landsat 4-5 protocol.

Select the option in the lower left corner Create the virtual raster of band set, to create the RGB, and then click on the Run key in the lower right corner to start the process.

Select the RGB folder within the Area-1 folder to save RGB.

Automatically the plugin merges the Bands, creating the RGB in natural color, Bands 1-2-3.

Select the RGB, click on it with the right mouse button, and open the RGB properties to turn it into a fake RGB color.

In the Red Band, Green Band, Blue Band, there are Bands 1-2-3 respectively, this combination and natural color, should change the Bands 1-2-3 by Bands 5-4-3 to obtain the false color.

In the Red Band put the Band-5.

In the Green Band put the Band-4.

And in the Blue Band put the Band-3.

Open the Load Min/Max Values window, select the Cumulative Count Cut option, and then Load.

To finish, click on Apply in the lower right corner of the window, and then click on OK.

The trimming RGB is in false color, with the combination of Bands 5-4-3 in that order.

This is the end of the first part of the image classification process, which was to download the treatment and cut of the study areas.

The next steps are related to the classification of land use and land cover.

11. Loading the SCP (Semi-Automatic Classification) plugin to start sorting

The SCP plugin has been used up to the present time only to treat and crop the images. Now the SCP plugin will be used to classify the land use and land cover.

To start loading the clippings of the area you want to sort, click on the 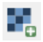 Raster key.

Open the Areas-1 folder, click on the RGB folder, and select the file, and Open.

With the RGB loaded, right click on the RGB button to open the properties, and change the Red Band that is with the Band-1 on the Band-5, the Green Band that has the Band-2 on the Band-4, the Blue Band that is with Band-3, with Band-3.

With the Bands changed, open the Load Min/Max Values window, select the Cumulative Count Cut option, click Load, Apply, and Ok. RGB is now in false color and loaded into the SCP plugin.

12. Getting Started

With RGB in fake color, open the SCP plugin tab in the lower left corner of the screen or in the upper toolbar on the SCP key.

Click on the Refresh list button on the top left side of the SCP plugin menu to load the RGB.

Open the window on the left side and select the RGB, which you want to sort.

With RGB selected, create a training set to start the classification. It can be created a new training by clicking on the key to the left of the menu of the plugin Create a new training input. If you already have a training set file, click on the Open training input button to open the existing training.

Click on the Create a new training input button to create a new training. Then, open the Area-1 folder, open the Training folder, and Save.

13. Starting the Training (making the signatures of the pixels)

With the RGB and Training loaded, open the Classification dock tab in the lower left corner.

The Info tab is Macro Class, MC ID is the Macro Class number, C Info is Class, and C ID is the class number.

Enter the Macro Class name in the MC Info field.

Macro Class 1 is Vegetation.

The Class is Preserved Forest.

The Activate ROI Pointer key serves to make a polygon automatically, simply clicking on any point in the image that automatically the system selects the pixels of the same value and forms a polygon.

The Create a ROI Polygon button is used to make a polygon manually.

Click on the button and click on a place in the area that corresponds to Preserved Forest. A polygon will form immediately, and then Click on the Save Temporary ROI to save the signature.

The process takes only a few seconds. When the signature is saved it appears in the frame of the ROI Signature list, on the upper left side.

Repeat as many times as necessary for each land use land cover category.

Change the Macro Class number in the MC ID field, place the number 2. For another Macro Class, change the Macro Class from Vegetation to Soil, and change the Preserved Forest Class to Exposed Ground.

Change Class and Macro Class, click on an area corresponding to Exposed Soil.

Click on the Save Temporary ROI to save the signature.

The process takes only a few seconds.

The signature has been saved and appears in the ROI Signature list box.

Repeat as many times as necessary.

Change the Exposed Soil class to another class corresponding to Ground, change it to Urban Ground.

Click on elsewhere in the area that corresponds to Urban Ground, and another signature will be generated.

Click on the Save Temporary ROI to save the signature.

The process takes only a few seconds.

The signature has been saved and appears in the ROI Signature list box.

Repeat as many times as necessary for each use or surface type.

Make the same for other land use and land cover categories, such as River.

Defining the colors of each class

Double-click on the color of each signature to be able to change them, leaving the signatures of the same class with the same color.

Open the core window and, for Preserved Forest, select the Green color and click on OK. Repeat the process with all the signatures of Preserved Forest.

Repeat the process with all other signatures.

Setting the colors of the Macro Class

Click on the Macroclasses tab, and change the colors of the Macros Classes according to what they suggest.

Double click on the color of the Macro Class to change it to Green Color (vegetation), Yellow Color (soil), and Blue Color (water).

Defining the algorithm for Classification

Open the Classification algorithm tab.

Select the type of algorithm to use for sorting, choose the Minimum Distance option.

14. Starting and Saving land use and land cover rating

Open the Classification output tab, select the Classification report option, and click on 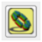 Run button to start sorting.

Select the Classification folder, and save it. The process will take a few seconds.

Ready sorting completed.

Click on the Layers tab, on the lower left side, and check the layers tab with the RGB Training and Classification.

Saving the rating

Click on the Save As button to save the rating.

Select the folder with the name of the location and the correct date, the area you have just classified, a name for the classification, and save it.

15. Quantifying land use and land cover

To start the quantification of land use and land cover, open the Classification folder and select the file with the extensions .qml and .Tif.

The classification file must be saved on the desktop.

Paste the files of the classification into the workspace, with the saved file. Rename the files, placing only a letter or number, avoiding big names, and then save them.

16. Transforming the Raster from sorting into a Shapefile

Click the Raster key to load the RGB to be sorted.

Load the sorted files that were previously saved.

At the top command prompt, click on Raster, go to Convert, Raster to Vector (Polygon).

Click on the select button in the Output File for Polygons (Shapefile).

Save these files to your desktop, then click Ok.

Automatically the Raster Transforms into a Shapefile (polygon).

Click on the New button to start a new assignment.

Go to the desktop and click on the folder Shapefile1, with the right button of the mouse.

Select the Area-1 folder, open the Shapefile folder, paste the Shapefile1 folder into this folder.

Click on it to verify that all files are correct, it should always contain 4 files with the following extensions .dbf, .prj, .shp, .shx.

Click on the Open button, choose the QGis folder, and open the folder of the location and date in question.

Click on and open the file that was previously saved, i.e, the file of the complete classification, the Shapefile of this area that was made must be saved together with these files; then click on the Vector key, to load the Shapefile under work.

The vector search window will open, click Search, open the Area-1 folder, open the Shapefile1 folder, and select the file with the .shp extension, click Open to load the Shapefile.

Right-click on the mouse for opening the properties of the Shapefile. In the Style tab open the first bar, Simple symbol.

It will open a window with several options. Choose “Categorized”, then click on the field of the Column below the previous one, and choose the 123 DN option.

Click Sort.The system will sort according to the information contained in the Shapefile.

Then, the colors of the classification should be the same as the signatures of the Shapefile.

Double click on the color to be changed; the color configuration window will open. Choose the coloration that matches the signature of the Shapefile, according to the classes, Preserved Forest, green; Exposed Soil, yellow; Urban Color, pink; and Blue Color, water.

It can be seen finally that the signatures of both the classification and the Shapefile are the same.

17. Loading Plugin Complement Statistic by Zone

Click on the button Statistics by Zone.

It will open a window already with some options selected. Make sure that Shapefile1 is in the field Polygonal layer containing the zones.

18. Loading the Group Stats plugin

Click on the GroupStats button.

It will open the GroupStats window.

Click on the option _count with the left mouse button and hold and drag to the second square on the right side of the Value window.

Now click on the option DN with the left mouse button and hold and drag to the second square on the left side of the Rows window.

Click on the option sum with the left mouse button and hold and drag to the second square on the right side of the Value window. With all the options loaded, click on Calculate.

The Plugin will calculate the pixel quantity of each signature, which will appear on the left side of the window.

19. Adding the signatures according to the color of each

Note that the Group Stats plugin resulting values will correspond to the signature number. The signatures 1-2-3 match Preserved Forest, whereas 4-5-9 Exposed Soil, 6-7-8 Urban Soil, 10 is for Rivers, and for Not Classified the value is 0.

Having these values in hand, you can move on to the next step which is to quantify these pixel values of each class by percentage.

20. Calculating the percentage of land use land cover

To know how much Preserved Forest has in this area, simply add the signatures of 1-2-3 = 2040 pixels, for Exposed Soil 4-5-9 = 2496 pixels, Urban Soil 6-7-8 = 749 pixels, and Rivers = 265 pixels. In some cases may appear a signature that was not made by the user, the own system classifies as an unclassified area that appears as a zero signature and its value is 75 pixels. The sum of all signatures equals 5625 pixels.

The total value of the pixels of each area will vary according to its size and the satellite. To transform these numbers of pixels in percentage, it is necessary to subtract from the total of pixels the values of zero signature and Rivers signature: 5625-75-265 = 5285 pixels.

Then the total number of pixels of the Preserved Forest is divided by the total value of the pixels already subtracted by unclassified and River signatures, multiplied by 100. This would be 2040 / 5285 * 100 = 38.60% of Preserved Forest. The same is applied to Exposed soil and Urban soil.

As a result, we estimate the percentage of each land use and land cover, as follows:

Preserved Forest = 38.60%

Exposed Soil = 47.23%

Urban Soil = 14.17%

21. Using the printing composer

The print composer is a tool for image saving and printing the data in QGis. First upload all the information (mount the map). The images can be saved in .png or .pdf extensions.

On the Tools Bar click on Project, select the New Print Composer option.

In the composer click on the Add New Map button and point the mouse cursor in the upper left corner, click on and pull to the lower right corner to determine the area represented by the red rectangle of the size of the image to be added.

To add the map scale click on the Add new scale bar button, then click on the map and the scale will automatically appear, open the item Properties tab, select the style of the scale, there are several scale style options, choose one, select Font and Colors, it will open the properties for the font and the color of the scale, click the Font button.

It will open a window where you can change the Font and Font Size.

In the Font color field, click on the color window on the right of the screen, and open the color panel; choose the best color for each map element.

To add a legend click on the Add New Legend button, just click anywhere on the map that the legend will appear automatically.

Organize the legend items; select the item and click on the keys to move the legend items to the desired position, organized items.

To add an image click on the Add Image button, and click on the map with the left button and define where the image will be included.

On the right side of the image has the properties of the image, click on the ... button to choose the desired image, open the folders to find the desired image, click on Open, the image will be loaded.

Select the Position and Size tab to define the size and position of the image; to change the size simply change the Width and Height fields; to change the positioning simply change the X and Y fields.

To add a title to the map click on the Add new label button, left-click on the map and drag by creating a rectangle to insert the title.

On the right side you will open the title properties, to place the desired title. In the Appearance tab, select Source to change the title properties.

Select the Position and Size tab for editing the title position; so, change the X, and Y fields to change the title position.

To save click on the Export as image button, choose the folder to save the image, then open a window to configure the image resolution, it is recommended to save as 300 dpi, and then click on Save.

To save in PDF click on the Export as PDF button, choose the folder to save the image to PDF.
